# The Impact of Ureteral Stent Indwelling Duration on Encrustation Degree and Extraction Difficulty: A Retrospective Study

**DOI:** 10.3390/jcm14124334

**Published:** 2025-06-18

**Authors:** Laurian Stefan Maxim, Ruxandra Maria Rotaru, Camelia Cornelia Scarneciu, Marius Alexandru Moga, Raul Dumitru Gherasim, Mihail Alexandru Badea, Alexandru Ghicavîi, Razvan Dragos Multescu, Bogdan Ovidiu Feciche, Ioan Scarneciu

**Affiliations:** 1 Emergency Clinical County Hospital Brasov, 500365 Brașov, Romania; maxim.laurian.stefan@unitbv.ro (L.S.M.); camelia.scarneciu@unitbv.ro (C.C.S.); mogas@unibv.ro (M.A.M.); vitalie.ghicavii@usmf.md (A.G.); ioan.scarneciu@unibv.ro (I.S.); 2 Faculty of Medicine, Transilvania University of Brasov, 500019 Brașov, Romania; 3 Department of Urology, University of Medicine, Pharmacy, Science and Technology “George Emil Palade” of Targu Mures, 540124 Targu Mures, Romania; raul-dumitru.gherasim@umfst.ro; 4 Natural Skin Târgu Mures, 540142 Targu Mures, Romania; mihail.badea@umfst.ro; 5 Department of Urology, “Saint John” Clinical Emergency Hospital, 042122 Bucharest, Romania; razvan.multescu@umfcd.ro; 6 Department of Urology, Emergency County Hospital Oradea, 410169 Oradea, Romania; feciche.bogdanovidiu@didactic.uoradea.ro

**Keywords:** ureteral stent, indwelling duration, encrustation, stent extraction, urological complications, urinary tract infections, polyurethane double-J stent, stent coloration, comorbidities

## Abstract

**Background/Objectives:** Ureteral stents are indispensable tools in contemporary urological practice; however, their prolonged indwelling is frequently associated with a spectrum of complications. This study aims to evaluate the correlation between indwelling duration and the extent of stent encrustation, as well as the impact on extraction difficulty. **Methods:** A retrospective analysis was conducted on 33 patients treated at Clinical County Emergency Hospital of Brașov between December 2023 and December 2024. All patients had polyurethane double-J ureteral stents placed. Parameters assessed included the degree of stent encrustation, discoloration, incidence of urinary tract infections (UTIs), and extraction difficulty. These were analyzed in relation to indwelling time and patient comorbidities. Statistical processing was performed using SPSS 23.0 software, with significance set at *p* < 0.05. **Results:** A statistically significant association was observed between longer stent indwelling times and higher grades of encrustation, particularly for the intervals of 45–90 days and over 90 days (*p* = 0.008 and *p* = 0.01, respectively). Low encrustation demonstrated correlations with certain comorbidities, whereas no statistically relevant associations were found for moderate and severe encrustation. Black coloration of the stents was strongly associated with UTIs caused by *Escherichia coli*, *Klebsiella* spp., and *Enterococcus* spp. (*p* < 0.001), as well as with extended indwelling durations (*p* < 0.001). No significant correlation was identified between the presence of UTIs and the degree of stent encrustation. **Conclusions:** Indwelling time is a critical determinant of both ureteral stent encrustation and discoloration, with direct implications for clinical decision-making regarding stent management and extraction planning. Timely removal and close monitoring are essential to reduce the risk of complications associated with long-term stent placement.

## 1. Introduction

Since 1967, ureteral stents have become essential tools in numerous urological procedures, playing a key role in the management of ureteral obstruction caused by a variety of factors, including ureteral stones, strictures, retroperitoneal tumors, fibrosis, and both endoscopic and surgical interventions on the ureter [1,2]. Advances in endoscopic techniques have significantly facilitated stent insertion, reducing the incidence of associated complications.

Complications related to ureteral stents may include discomfort, urinary tract infections, and stent migration. These issues have driven the development of advanced stent designs equipped with anchoring systems or biodegradable materials. However, serious complications can arise when stents are left in place for extended periods, including migration, stone formation, and stent fragmentation [3,4]. The incidence of stent encrustation increases with the duration of indwelling time [5].

Although modern stents are equipped with hydrophilic coatings, the precise etiology of encrustation remains unclear, despite continuous improvements in stent materials and properties. The objective of this study was to assess the association between ureteral stent indwelling time and encrustation level, as well as the procedural difficulty of stent extraction [6].

## 2. Materials and Methods

A retrospective study was conducted in the Urology Department of the Clinical County Emergency Hospital of Brașov over a 12-month period, from December 2023 to December 2024. The study included a cohort of 33 patients who underwent ureteral stent placement either as an emergency procedure (for obstructive ureteral lithiasis) or electively (for ureteral strictures or retroperitoneal fibrosis). Only patients with neglected ureteral stents or stents associated with complications were included. All patients underwent preoperative imaging investigations, such as computed tomography or plain radiography.

The stents used were double-J type, with calibers ranging from 4.7 to 7 Fr.

All patients were adequately informed and provided written informed consent for each surgical procedure, acknowledging the associated risks. The subsequent study conducted on the extracted ureteral stents posed no risk to the participants.

Stent removal was performed under general or spinal anesthesia. The level of extraction difficulty was assessed based on the procedure employed, which included flexible ureteroscopy with laser lithotripsy, fragmentation of calculi located at the distal coil of the stent, or cystoscopy. Extraction procedures were classified as moderately difficult or impossible.

Intraoperative evaluation of stent encrustation was performed by introducing a hydrophilic guidewire with a diameter of 0.035 cm. A “moderate” degree of encrustation was assigned when the guidewire could pass through (for low and moderate incrustation); complete encrustation was defined by the inability to advance the wire (for severe incrustation). After removal, the stents were photographed using macro lenses, and the images were analyzed using specialized software to quantify the degree of encrustation.

The extracted stents were also classified according to color into three groups: group I: stents with no color changes; group II: brown-colored stents; and group III: black-colored stents.

Statistical analysis was performed using SPSS 23.0 software. The chi-square test, Kendall’s tau, and Spearman’s rank correlation coefficient were utilized to assess the data. A *p*-value < 0.05 was considered statistically significant.

## 3. Results

### 3.1. Patient Demographics and Clinical Characteristics

The study included a total of 33 patients enrolled between December 2023 and December 2024, who underwent ureteral stent placement either as an emergency procedure for obstructive urolithiasis or electively for chronic conditions such as oncological diseases and retroperitoneal fibrosis. The study was conducted with a small sample size, which limits its statistical power and increases the likelihood that its findings are due to chance rather than true effects (Table 1).

The mean age of the patients was 49.24 years. Regarding sex distribution, there was a predominance of male patients, with 19 men and 14 women included in the study.

The most frequently encountered comorbidities were arterial hypertension (33.3%) and diabetes mellitus (18.2%). Other comorbidities included obesity (12.1%), oncological conditions (6.1%), and retroperitoneal fibrosis (3%). Additionally, four patients were receiving anticoagulant therapy. Only five patients had no associated comorbidities.

Patients were categorized into four groups according to the stent’s intended duration and the actual indwelling period. Among those with stents designed for a 3-month duration, six patients had stents in place for less than 45 days, fourteen patients had stents for a period between 45 and 90 days, and nine patients had stents retained for more than 90 days. The latter were classified as having neglected ureteral stents. The fourth group included four patients who received 12-month stents due to chronic conditions that required urinary tract decompression, such as ureteral strictures or retroperitoneal fibrosis.

Although two patients had bilateral ureteral stents, only one procedure per patient was considered in the analysis for consistency.

### 3.2. Characteristics of Ureteral Stents

The material used to produce the ureteral stents was polyurethane. A total of 33 stents were used, each measuring 26 cm in length, with diameters ranging from 4.7 Fr to 7 Fr. Ureteral stents with calibers of 5 Fr, 8 Fr, or 10 Fr were not employed in this study (Table 2).

The incidence of stent placement was higher on the left side compared to the right (21 versus 12 cases, respectively). The indication for ureteral stent insertion was either emergency intervention in cases of obstructive lithiasis or elective placement in patients with retroperitoneal fibrosis or oncological conditions causing ureteral stenosis.

The correlation between the degree of encrustation and the associated pathologies reported and recorded in this study (obesity, retroperitoneal fibrosis, and under antiplatelet treatment) showed statistically significant results in cases of low encrustation (*p* < 0.001). However, no statistically significant correlation was observed in cases of moderate or severe encrustation (Table 3).

Moreover, the presence of associated pathologies was found to promote the occurrence of urinary tract infections, particularly in cases involving *E. coli* (*p* < 0.05), *Klebsiella*, and *Enterococcus* species (*p* < 0.001). Additionally, stent discoloration, specifically black pigmentation, was significantly associated with the presence of these pathogens, with statistically significant results (*p* < 0.001) (Table 4 and Table 5).

Regarding the caliber of the ureteral stents, statistically significant results were observed in association with black discoloration across all sizes used (4.7 Fr, 6 Fr, and 7 Fr) (*p* < 0.001). Low encrustations were recorded for all calibers as well, with statistically significant findings (*p* < 0.001) (Table 6).

The indwelling duration of ureteral stents influences the occurrence of low encrustations. Among patients who received stents with a 3-month intended duration, a significant positive association was observed between low encrustation and stent retention for 45–90 days (*p* = 0.008) as well as for more than 90 days (*p* = 0.01) (Table 7).

No statistically significant correlations were identified between the presence of urinary tract infections and the degree of ureteral stent encrustation.

There were no statistically significant differences regarding brown stent discoloration among patients with stents retained for less than 45 days, stents retained for 45–90 days, 1-year indwelling stents, or neglected stents.

However, statistically significant differences were found between the three stent indwelling duration groups (<45 days, 45–90 days, >90 days) and the presence of black discoloration. As the duration of stent retention increased, black discoloration became more frequent.

A statistically significant difference was observed between patients with stents retained for less than 45 days and those with stents retained for 350–366 days, with black discoloration being more frequently found in the latter group (*p* = 0.005 *).

Additionally, statistically significant differences were recorded between patients with stents retained for 45–90 days and those with 350–366-day stents in terms of black discoloration (*p* < 0.001 **).

Furthermore, a significant difference was observed between patients with stents retained for 350–366 days and those with stents retained for more than 90 days, with black discoloration being less frequently observed in the latter group compared to the former (*p* = 0.01 *) (Table 8).

The type of surgical intervention required was closely associated with the duration of ureteral stent indwelling. Patients with stents maintained for longer periods, particularly those exceeding 90 days or those classified as neglected, more frequently required complex surgical procedures such as flexible ureteroscopy with laser lithotripsy or combined endourological approaches. In contrast, patients with stents retained for less than 45 days most commonly underwent standard cystoscopic removal, with minimal need for adjunctive surgical maneuvers. These findings suggest that prolonged stent retention increases the likelihood of requiring more invasive or technically demanding surgical interventions.

In the patient group with ureteral stents retained for less than 45 days, the most frequently associated interventions during stent removal were distal loop fragmentation and rigid or flexible ureteroscopy. Among patients with stents retained for 45–90 days, ureteroscopy (URS/FURS) was performed in 71.4% of cases, while in the neglected stent group (>90 days), it was required in 80% of cases. In contrast, for patients with long-term indwelling stents (validity of 1 year), this intervention was necessary in only 33.3% of cases.

Distal loop fragmentation was performed in 50% of patients in the <45-day group, in 42.9% of those in the 45–90-day group, and in 40% of patients with neglected stents (>90 days).

Ureteral stents were subsequently removed either by cystoscopy or ureteroscopy, depending on the complexity of encrustation and the surgical approach required (Table 9).

The localization of encrustations was found to vary significantly depending on the duration of stent indwelling. Encrustations at the distal loop of the stent were identified in 83% of patients with stents retained for less than 45 days, in 92% of those with stents maintained for 45–90 days, and in 80% of patients with neglected stents (>90 days). In contrast, only 33% of patients with long-term stents (valid for up to one year) presented with encrustations at the distal loop.

Encrustations located along the body of the stent were observed in 50% of cases with neglected stents. Proximal loop encrustation was recorded in 70% of these patients, indicating a higher degree of overall stent colonization in prolonged indwelling scenarios.

These findings suggest that shorter indwelling times tend to result in encrustations limited to the distal segment, whereas neglected stents are more frequently associated with extensive, multi-site encrustations involving the shaft and proximal loop.

## 4. Discussion

Auge BK and colleagues highlighted that ureteral stent encrustations can begin to form as early as two weeks post-insertion [7]. Our findings are consistent with this observation, demonstrating that encrustations were present even in stents maintained for less than 45 days. Within this group, one patient exhibited low encrustation, four patients showed moderate encrustation, and one case presented with severe encrustation.

Among patients with stents retained for 45 to 90 days, low encrustations were identified in two cases, moderate encrustations in five cases, and severe encrustations in seven cases. In the group with neglected stents (indwelling time > 90 days), two patients had severe encrustations, while one patient had moderate encrustation.

These results reinforce the existing literature suggesting that encrustation is a time-dependent phenomenon. However, our data also emphasize that encrustation can occur even during short-term use, indicating that additional factors such as comorbidities, infections, and stent material may contribute to the development and severity of encrustation.

Another study conducted by Burgos R et al. demonstrated that encrustations significantly reduce intraluminal flow, which appears to be more critical than the mere presence of the deposits themselves [8]. This reduction in flow may result in serious complications, such as urinary tract obstruction and compromised renal function [9]. These findings underscore the clinical importance of early detection and timely removal or replacement of ureteral stents to prevent adverse outcomes.

In a study conducted by el-Faqih Sr et al., the rate of ureteral stent encrustation increased significantly with longer indwelling times, rising from 9.2% for stents retained for up to 6 weeks to 47.5% for those between 6 and 12 weeks, and reaching 76.3% for stents left in place for more than 12 weeks [10]. In our study, low encrustations were observed across all stent calibers, regardless of the indwelling time. However, prolonged stent placement remains a well-established risk factor for encrustation development [11,12], further supporting the necessity for regular follow-up and timely intervention.

The presence of urinary tract infections (UTIs) has been shown to significantly influence the process of stent encrustation [13]. Consequently, early removal of ureteral stents is recommended in patients with documented infections [14], as well as in those with underlying metabolic disorders that promote urinary stone formation [5,9,10]. It is hypothesized that the rapid development of encrustations in these patients is linked to the mineralization of bacterial biofilm on the stent surface [15]. However, in our study, no statistically significant correlations were observed between the presence of UTIs and the degree of encrustation.

Additionally, the material composition of ureteral stents plays a pivotal role in the rate of encrustation. As reported by Cauda et al., stents made from polyurethane are more susceptible to encrustation [16], which aligns with our findings—where all patients with polyurethane stents exhibited varying degrees of encrustation (low, moderate, or severe). Notably, low encrustation was recorded across all stent diameters used in our cohort (4.7 Fr, 6 Fr, and 7 Fr) [17].

Kawahara and colleagues identified the coloration of ureteral stents in the presence of urinary tract infections caused by hydrogen sulfide-producing bacteria (*Escherichia coli* and *Klebsiella pneumoniae*) [17,18,19]. This finding was also observed in our study, particularly in cases where black coloration of the stent was noted.

Regarding the extraction procedures, for stents that exhibited encrustation at the distal coil regions, mechanical lithotripsy or laser fragmentation of the stones was employed. In cases where encrustation occurred at the body of the stent or the proximal coil, rigid or flexible ureteroscopy with holmium laser fragmentation or pneumatic lithotripsy was used [20,21,22].

### Limitations of the Study

This study has several limitations. Definitive conclusions cannot be drawn in the absence of a comparison group, and the small sample size limits both the statistical power and the generalizability of the results. Furthermore, no power analysis was performed to determine the appropriate number of participants required to detect statistically significant differences.

## 5. Conclusions

The results of this study highlight the significant impact of various factors on the occurrence and severity of encrustations in ureteral stents. The duration of stent retention plays a key role, with prolonged periods of stent indwelling (over 45 days) being strongly associated with low encrustation, particularly in patients with stents neglected for over 90 days. The presence of urinary tract infections, particularly with hydrogen bacteria like *Escherichia coli* and *Klebsiella* pneumoniae, was correlated with black coloration of the stents, which was also found to be a strong indicator of encrustation.

Furthermore, encrustation was observed across all stent diameters used, emphasizing that the material composition of polyurethane stents is prone to biofilm formation, leading to varying degrees of encrustation. In terms of clinical management, the use of ureteroscopy combined with laser fragmentation or pneumatic lithotripsy was essential for stent removal, depending on the location and extent of encrustation.

The findings from this study contribute to a deeper understanding of the factors influencing ureteral stent encrustation and the challenges posed by long-term stent retention, providing valuable insights for improving patient care and stent management strategies. Further research is warranted to explore the potential benefits of earlier stent removal, as well as the development of stent materials with improved resistance to encrustation.

## Figures and Tables

**Table 1:** General characteristics.

		Patients	Percentage
Age (mean +/− standard deviation)		M 49.24 SA 15.66 R 23–87	-
Gender	Male	19	57.6
	Female	14	42.4
Comorbidities	Hypertension	11	33.3
	Diabetes	6	18.2
	Oncological disease	2	6.1
	Obesity	4	12.1
	Retroperitoneal fibrosis	1	3
	Antiplatelet treatment	4	12.1
	No disease	5	15.15

**Table 2:** Ureteral stents characteristics.

Variables		Number	Percentage
Indication	Emergency (lithiasis)	13	38.4%
	Chronic (replacement/strictures)	20	60.6%
Availability	3 months	29	87.9%
	12 months	4	12.1%
Indwelling time	days	147.7	Range: 21–583 SD: 144.4
Stent length (cm)	cm	26	
Stent caliber (F)	4.7	6	18.2%
	6	20	60.6%
	7	7	21.2%
Side	Left	21	63.6%
	Right	12	36.4%

**Table 3:** UTI presence and stent coloration.

	Color	Brown			Black		
		*n*	Result	*p*-Value	*n*	Result	*p*-Value
UTI	*E. coli*	9	χ^2^ (1) = 6.81 *	*p* < 0.05	9	χ^2^ (1) = 6.81 *	*p* < 0.05
	*Klebsiella*	2	χ^2^ (1) = 25.48 **	*p* < 0.001	2	χ^2^ (1) = 25.48 **	*p* < 0.001
	*Enterococcus*	5	χ^2^ (1) = 16.03 **	*p* < 0.001	5	χ^2^ (1) = 16.03 **	*p* < 0.001

Note: *n* = 33 patients, * *p* < 0.05, ** *p* < 0.001.

**Table 4:** Encrustation grade and ureteral stent caliber.

Encrustation	Low		Moderate		Severe	
Caliber	Result	*p*-Value	Result	*p*-Value	Result	*p*-Value
4.7	χ^2^ (1) = 18.939 **	*p* < 0.001	χ^2^ (1) = 2.455	*p* = 0.117; *p* > 0.05	χ^2^ (1) = 0.030	*p* = 0.862; *p* > 0.05
6	χ^2^ (1) = 18.939 **	*p* < 0.001	χ^2^ (1) = 2.455	*p* = 0.117; *p* > 0.05	χ^2^ (1) = 0.030	*p* = 0.862; *p* > 0.05
7	χ^2^ (1) = 18.939 **	*p* < 0.001	χ^2^ (1) = 2.455	*p* = 0.117; *p* > 0.05	χ^2^ (1) = 0.030	*p* = 0.862; *p* > 0.05

Note: *n* = 33 patients, ** *p* < 0.001.

**Table 5:** Coloration and ureteral stent caliber.

Color	Brown		Black	
Caliber	Result	*p*-Value	Result	*p*-Value
4.7	χ^2^ (1) = 3.667	*p* = 0.056; *p* > 0.05	χ^2^ (1) = 16.030 **	*p* < 0.001
6	χ^2^ (1) = 3.667	*p* = 0.056; *p* > 0.05	χ^2^ (1) = 16.030 **	*p* < 0.001
7	χ^2^ (1) = 3.667	*p* = 0.056; *p* > 0.05	χ^2^ (1) = 16.030 **	*p* < 0.001

Note: *n* = 33 patients, ** *p* < 0.001.

**Table 6:** Encrustation grade and indwelling time.

	Encrustation	Low			Moderate			Severe		
	Patients	*n*	Result	*p*-Value	*n*	Result	*p*-Value	*n*	Result	*p*-Value
<45 d	6/33	1	χ^2^ (1) = 2.66	*p* = 1.10	4	χ^2^ (1) = 0.66	*p* = 0.41	1	χ^2^ (1) = 2.66	*p* = 0.10
45–90 d	14/33	2	χ^2^ (1) = 7.14 *	*p* = 0.008	5	χ^2^ (1) = 1.14	*p* = 0.28	7	χ^2^ (1) = 0.00	*p* = 1
>90 d	10/33	1	χ^2^ (1) = 6.40 *	*p* = 0.01	2	χ^2^ (1) = 3.60	*p* = 0.058	7	χ^2^ (1) = 1.60	*p* = 0.20
1 y	3/33	0	--	-	1	χ^2^ (1) = 0.33	*p* = 0.56	2	χ^2^ (1) = 0.33	*p* = 0.56

Note: *n* = 33 patients, * *p* < 0.05.

**Table 7:** Encrustation grades and UTI.

	Encrustation	Low		Moderate		Severe	
		Result	*p*-Value	Result	*p*-Value	Result	*p*-Value
UTI	*E*. *coli*	-	*p* > 0.05	χ^2^ (1) = 0.349	*p* > 0.05	-	*p* > 0.05
	*Klebsiella*	-	*p* > 0.05	-	*p* > 0.05	-	*p* > 0.05
	*Enterococcus*	-	*p* > 0.05	-	*p* > 0.05	-	*p* > 0.05

Note: *n* = 33 patients.

**Table 8:** The type of surgical intervention associated with the duration of ureteral stent indwelling.

		Fragmentation on Distal		URS + FURS		Cystoscopy	
	Patients	*n*	%	*n*	%	*n*	%
<45 d	6/33	3	50	3	50	1	16.7
45–90 d	14/33	6	42.9	10	71.4	14	100
>90 d	10/33	4	40	8	80	1	10
1 y	3/33	1	33.3	1	33.3	1	33.3

**Table 9:** Association between stent indwelling time and encrustation site.

	Encrustation	Distal		Body		Proximal	
	Patients	*n*	%	*n*	%	*n*	%
<45 d	6/33	5	83.3	2	33.3	3	50
45–90 d	14/33	13	92.9	2	14.3	7	50
>90 d	10/33	8	80	5	50	7	70
1 y	3/33	1	33.3	1	33.3	2	66.7

## Data Availability

The original contributions presented in this study are included in the article. Further inquiries can be directed to the corresponding author.

## Abbreviation

The following abbreviation is used in this manuscript:

UTI	Urinary tract infection
